# High-Performance Flexible Supercapacitors obtained via Recycled Jute: Bio-Waste to Energy Storage Approach

**DOI:** 10.1038/s41598-017-01319-w

**Published:** 2017-04-26

**Authors:** Camila Zequine, C. K. Ranaweera, Z. Wang, Petar R. Dvornic, P. K. Kahol, Sweta Singh, Prashant Tripathi, O. N. Srivastava, Satbir Singh, Bipin Kumar Gupta, Gautam Gupta, Ram K. Gupta

**Affiliations:** 1 Department of Chemistry, Pittsburg State University, 1701 S. Broadway, Pittsburg, Kansas 66762 USA; 2 Department of Physics, Pittsburg State University, 1701 S. Broadway, Pittsburg, Kansas 66762 USA; 3 Department of Physics, Banaras Hindu University, Varanasi Uttar Pradesh, 221004 India; 4 CSIR -National Physical Laboratory, Dr. K.S. Krishnan Road, New Delhi, 110012 India; 5 Materials Physics and Applications (MPA-11), Los Alamos National Laboratory, Los Alamos, New Mexico 87545 USA

## Abstract

In search of affordable, flexible, lightweight, efficient and stable supercapacitors, metal oxides have been shown to provide high charge storage capacity but with poor cyclic stability due to structural damage occurring during the redox process. Here, we develop an efficient flexible supercapacitor obtained by carbonizing abundantly available and recyclable jute. The active material was synthesized from jute by a facile hydrothermal method and its electrochemical performance was further enhanced by chemical activation. Specific capacitance of 408 F/g at 1 mV/s using CV and 185 F/g at 500 mA/g using charge-discharge measurements with excellent flexibility (~100% retention in charge storage capacity on bending) were observed. The cyclic stability test confirmed no loss in the charge storage capacity of the electrode even after 5,000 charge-discharge measurements. In addition, a supercapacitor device fabricated using this carbonized jute showed promising specific capacitance of about 51 F/g, and improvement of over 60% in the charge storage capacity on increasing temperature from 5 to 75 °C. Based on these results, we propose that recycled jute should be considered for fabrication of high-performance flexible energy storage devices at extremely low cost.

## Introduction

Supercapacitors are electrochemical energy storage devices primarily attractive for their fast charging and discharging capability, long lasting stability and safe handling^1, 2^. Because of these unique qualities, supercapacitors are ideal candidates for a number of applications including regenerative breaking in cars, static random access memory and motor starters^3, 4^. In supercapacitor devices, electrode materials are separated by an ion transport layer through which electrolyte ions shuttle to the electrode surfaces during the charging and discharging processes^5^. Based on their utilities, the electrolytes widely vary from aqueous to non-aqueous and from liquid to solid, but the materials that function as electrodes are the key determiners of supercapacitors’ performance. To date, three main material classes, based on their charge storage mechanism, have been investigated for electrode applications^3^. These include carbonaceous materials, metal oxides and polymers or their composites^6–17^. Metal oxide based supercapacitors have high capacitance, but severely suffer in stability, making carbonaceous materials that store charges through double layer adsorption mechanism particularly attractive^11, 17, 18^. In addition, carbonaceous materials are relatively non-toxic, chemically inert, stable at high temperatures, easily accessible and often sustainable^19–21^. Among them, different forms of carbon including carbon nanotubes, graphene, graphite, activated carbons, carbon nanofibers and carbon aerogel have been attracted particular attention due to their high surface area and high porosity^9, 22–25^.

Production of activated carbon by pyrolysis of biological materials (biomass) followed by chemical or physical activation for use as a supercapacitor electrodes seems especially attractive since it addresses waste handling issues, offers cheaper form of recycling method and reduces land-fill^26, 27^. However, while each biomass has distinctive intrinsic structure that dictates its property, there is still no useful method to predict electrochemical behaviour of carbon that would result from such a resource without actually performing experiments. Hence, this lack of the prediction tool necessitates experimental investigation of electrochemical properties of biomass derived carbons from different sources separately, and as a consequence a number of biomass materials have been studied including plant-based sugar cane bagasse, apple pulp, cherry stones, rice husk, coffee shells, potato starch, bamboo, walnut shell and pistachio shell and animal based chicken egg shell membranes^28–33^. However, studies on flexible supercapacitor devices and effect of temperature on their charge storage capacity are still limited.

Here, we report on the first electrochemical investigation of a new type of jute-derived carbon electrodes and supercapacitor devices made from them. We chose jute for our investigation because of its intrinsic fibrous structure which is expected to be beneficial for ionic conductivity, and because jute is the second most popular natural fiber in the world. It is composed primarily of cellulose and lignin, and it has been used for centuries for ropes, sacking, carpets etc. Our investigations revealed that jute-derived carbon electrodes provide specific capacitance of 408 F/g at 1 mV/s, with 100% retention of charge storage capacity during cyclic and bending tests. The supercapacitor device fabricated from these electrodes gave specific capacitance of 51 F/g at 5 mV/s, which is among the top reported values for such devices (Table 1). Moreover, the device also showed over 60% improvement in charge storage capacity on increasing temperature from 5 to 75 °C.

**Table 1:** Electrochemical properties of KOH activated bio-mass.

Material	Max. Capacitance (F/g)	Electrochemical testing performed under	Reference
Bamboo	66	5 mV/s	46
Bamboo	68	1 mA/cm^2^	47
Firwood	138	3 mA/cm^2^	48
Cassava peel waste	153	—	49
Recycled waste paper	180	2 mV/s	50
Cherry stones	230	1 mA/cm^2^	51
Corn grains	257	1 mA/g	52
Pistachio shell	261	200 mA/g	29
pitch	267	50 mA/g	36
Sunflower seed shell	311	250 mA/g	40
Potato starch	335	50 mA/g	42
Arganseed shells	355	125 mA/g	53
**Recycled jute**	**408**	1 mV/s	**This work**
	**185**	500 mA/g	

## Experimental

**Figure 1:**
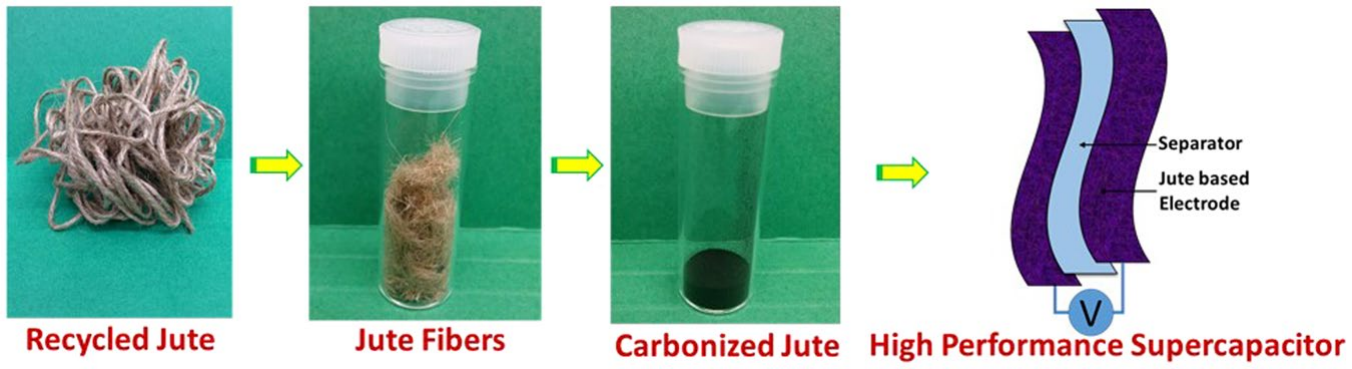
Schematic of the device fabrication process.

Figure 1 shows a schematic of the process used for preparation of carbonized jute fibers in this work. Jute rope was purchased from Walmart and untangled to obtain jute fibers. One gram of such fibers was hydrothermally treated at 180 °C for 24 hours in 30 ml 1 M H_2_SO_4_ solution. After cooling to room temperature, the fibers were washed and dried at 70 °C overnight. The pre-treated jute fibers were chemically activated by heating with KOH (1:1 ratio of pre-treated jute and KOH) at 800 °C (3 °C/min) for 1 hour under an argon atmosphere. After cooling back to room temperature, the obtained carbonized jute was washed with 1 M HCl and deionized (DI) water, and dried in an oven for 12 hours at 70 °C, before testing and supercapacitor device fabrication.

The carbonized jute was structurally and electrochemically characterized using various techniques. X-ray diffraction (XRD), Raman spectroscopy, scanning electron microscopy (SEM) and transmission electron microscopy (TEM) were used for structural characterizations of carbonized jute. The XRD spectra of the sample was taken using Shimadzu X-ray diffractometer using the 2*θ* − *θ* scan with CuK_α1_ (λ = 1.5406 Å) radiation. Raman studies were carried out using an argon ion laser at a wavelength of 514.5 nm as the excitation source (Model Innova 70, Coherent). Scanning electron microscope imaging was carried out using a JEOL 7000 FE SEM equipped with electron backscatter diffraction (EBSD), secondary electron (SE), backscattered electron (BE) and transmission electron (TE) detectors. TEM imaging was performed using a FEI-Tecnai, 200 kV transmission electron microscope equipped with a CCD camera for STEM, HAADF detector, and EDX. TEM image non-linear processing was carried out using Gatan digital micrograph version 3.4. The surface area was determined by the Brunauer–Emmett–Teller (BET) adsorption method (Micrometrics, USA, ASAP 2020 Models). A jute sample was first degassed for 24 hours at a holding temperature of 90 °C, following which the analysis for nitrogen adsorption was done at liquid nitrogen temperature (−196 °C).

The charge storage capacity and other electrochemical properties were studied using a VersaSTAT 4-500 electrochemical workstation (Princeton Applied Research, USA). For electrochemical measurements, a three-electrode system consisting of a platinum wire counter electrode, saturated calomel reference electrode and jute fibers on nickel foam as a working electrode was used. All electrochemical testing was performed in 3 M KOH electrolyte. The working electrode was prepared by mixing 80 wt.% of carbonized jute fibers, 10 wt.% of acetylene black and 10 wt.% of polyvinylidene difluoride (PVdF) in the presence of N-methyl pyrrolidinone (NMP). After thoroughly mixing, the paste was applied onto pre-cleaned nickel electrode, and the loading of carbonized jute was measured by weighing the nickel foam before and after electrode preparation using an analytical balance (model MS105DU, Mettler Toledo, max. 120 g, 0.01 mg of resolution). The mass loading of carbonized jute was around 3–4 mg/cm^2^. Supercapacitor devices were fabricated using the above described working electrodes and ion transporting separator. Electrochemical properties of the electrode and supercapacitor device were investigated using cyclic voltammetry (CV), galvanostatic charge-discharge and electrochemical impedance spectroscopy (EIS) methods. EIS studies were performed over a frequency range of 0.05–10 kHz at open circuit potential.

## Results and Discussions

**Figure 2:**
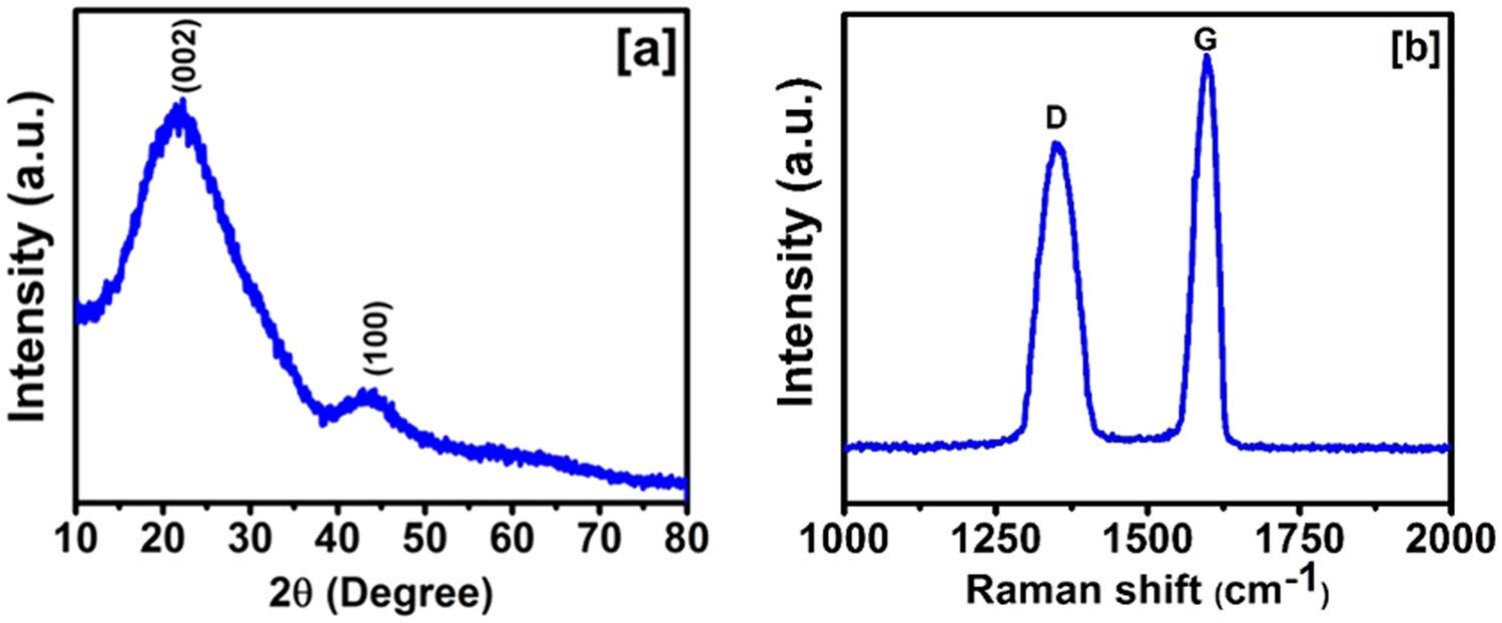
(**a**) XRD patterns and (**b**) Raman spectrum of the carbonized jute.

The structural and electrochemical properties of the carbonized jute fibers were studied in details. The X-ray diffraction (XRD) pattern of the carbonized jute is shown in Fig. 2a. The XRD patterns confirm the presence of graphitic phase of the carbon. The peaks at 21.9 and 43.7 degree were assigned as (002) and (100) indices, respectively as the graphitic carbon. The peak at 43.7 degree indicates the formation of a higher degree of intralayer condensation of graphite^6^. The graphitic phase of carbon is conducting form of carbon expected to be beneficial in reducing resistance during electrochemical process. The presence of graphitic phase was further confirmed by Raman spectroscopy. The Raman spectrum of the carbonized jute in Fig. 2b shows bands at 1353 cm^−1^ and 1597 cm^−1^ corresponding to diamond (D) and graphite (G) phases of carbon^34^. The ratio of intensities of D and G bands provides disorder information of carbon. From the spectrum, it can be seen that the intensity of G band (I_G_) is higher than D band intensity (I_D_) suggesting good structural alignment. For the carbonized jute, a ratio of 1.08 was observed for G/D band intensities. Additionally, it is worth noting that the I_G_/I_D_ value is in between 0.52 of commercial activated carbons and ordered carbon nanosheets^35^, wherein the previously reported ratios go close to 0.90 for similar pyrolysis temperatures^34^. Recently, a hierarchical porous carbon was synthesized using sulfonated pitch as a precursor in a simple KOH activation process^36^. The effect of KOH to sulfonated pitch ratio on the porosity and the specific surface area was reported and it was observed that the D band intensity increased with the KOH to sulfonated pitch ratio. As a consequence of increase in D band intensity, the electrical conductivity decreased and thus the charge storage capacity.

**Figure 3:**
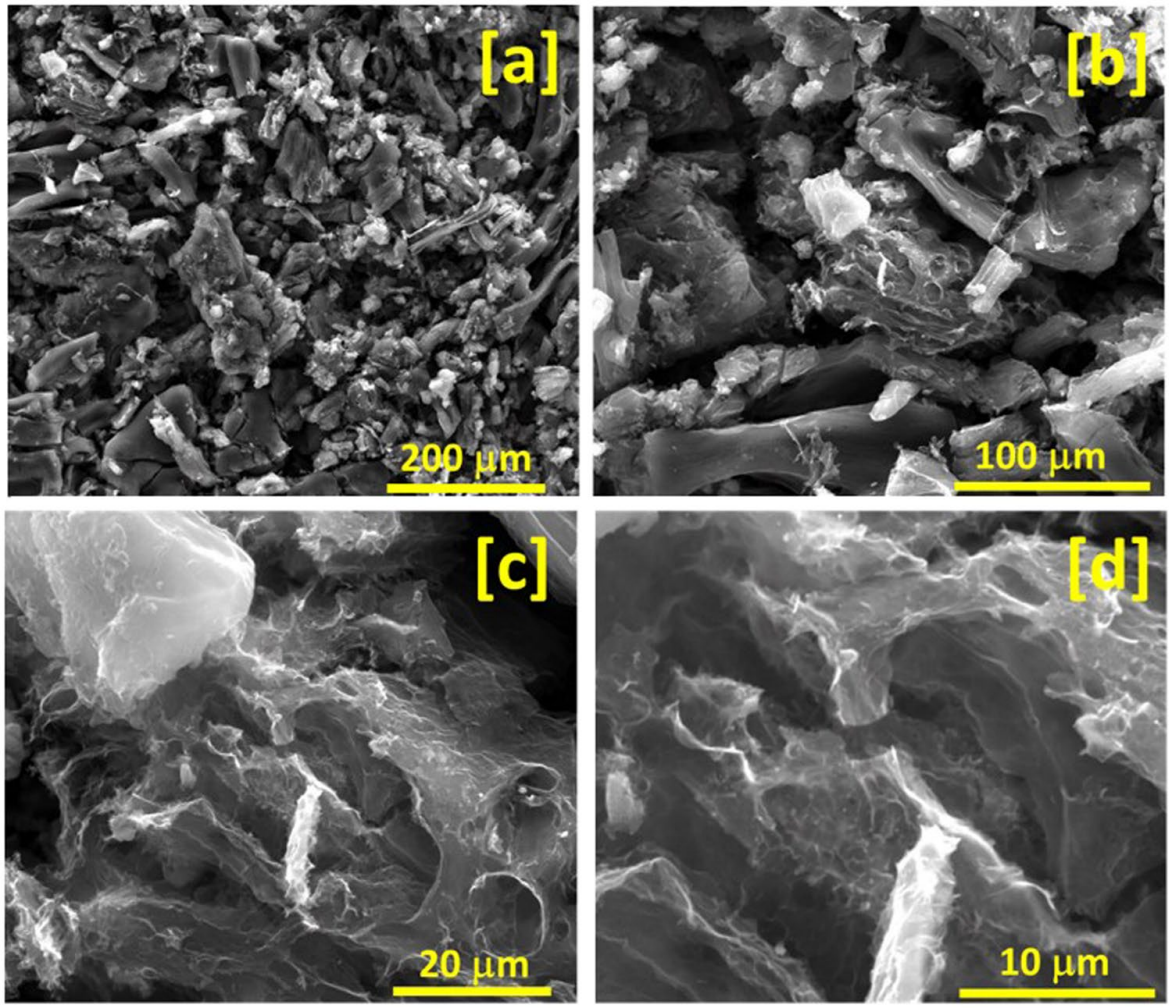
SEM images of the carbonized jute at various magnifications.

BET nitrogen adsorption/desorption isotherm measurements were used to determine the surface area and porosity of the jute-derived carbon. Figure S1a (ESI) shows adsorption/desorption isotherm with a slight hysteresis loop characteristic of a type IV isotherm. The hysteresis loop in the isotherm is the indication of capillary condensation, which arises due to different mechanisms of adsorption/desorption in micropores. As hysteresis is observed in the low relative pressure range of the isotherm plot that confirms the presence of micropores in the system. The carbonized jute has very high specific surface area of 1769 m²/g. BET isotherm plot confirms the microporous nature of the sample, which is also proven by pore size distribution (PSD) from BJH (Barrett-Joyner-Halenda) adsorption plot shown in Fig. S1b (ESI). The PSD plot for jute as obtained by N_2_ adsorptions basically shows a bimodal distribution of micropores and mesopores. From the PSD plot, it is found that maximum pores in the sample have the diameter of 1.7 nm with the average pore diameter of 2.9 nm. Several other related reports are worth mentioning. Zhang *et al*. steam-activated poplar bark to prepare activated carbon with high surface area^37^. They studied the effect of activation temperature on the surface area of the polar bark and observed that below 800 °C the adsorbed volume of nitrogen was around 200 cm^3^/g. The adsorbed volume at 900 °C was increased to about 400 cm^3^/g with well-developed micro- pores. Kumagai *et al*. produced micro- and mesoporous activated carbon from a mixture of rice husk and beet sugar as a precursor^38^. Their product had a BET specific surface area of 1357 m^2^/g with total pore volume of 0.99 mL/g. He *et al*. reported on using ZnCl_2_ as an activating agent to produce mesoporous carbons with high surface area (1527–1634 m^2^/g) for supercapacitor applications from peanut shell and rice husk^39^, while Li *et al*. investigated the effect of activation temperature and concentration of activating agent on the surface area and porosity of activated carbon from sunflower seed shell^40^. They also found that BET surface area and total pore volume increased sharply with the increase of activation temperature from 600 to 700 °C, but levelled off when activation temperature was higher than 700 °C. It was further noticed that at the same activating temperature, higher concentration of KOH as activating agent resulted in higher BET surface area and total pore volume. Further confirmation of high surface area and microstructure of our carbonized jute was obtained by SEM and TEM studies. Figure 3 shows the SEM images of the carbonized jute. The higher magnification images reveal that carbonized jute has high surface area. The carbonized jure showed micro-porous structure. Porosity is beneficial for electrodes in energy storage devices since it provides for high surface area. The energy storage process in electrodes from carbonized bio-mass is due to formation of electrochemical double layer which increases with increase in surface area. In our process, the pores originate from activation of the jute with KOH through a reaction that can be represented by the following reaction:

1 $$3\,KOH+C\,\to {K}_{2}C{O}_{3}+\,{K}_{2}O+2{H}_{2}$$

**Figure 4:**
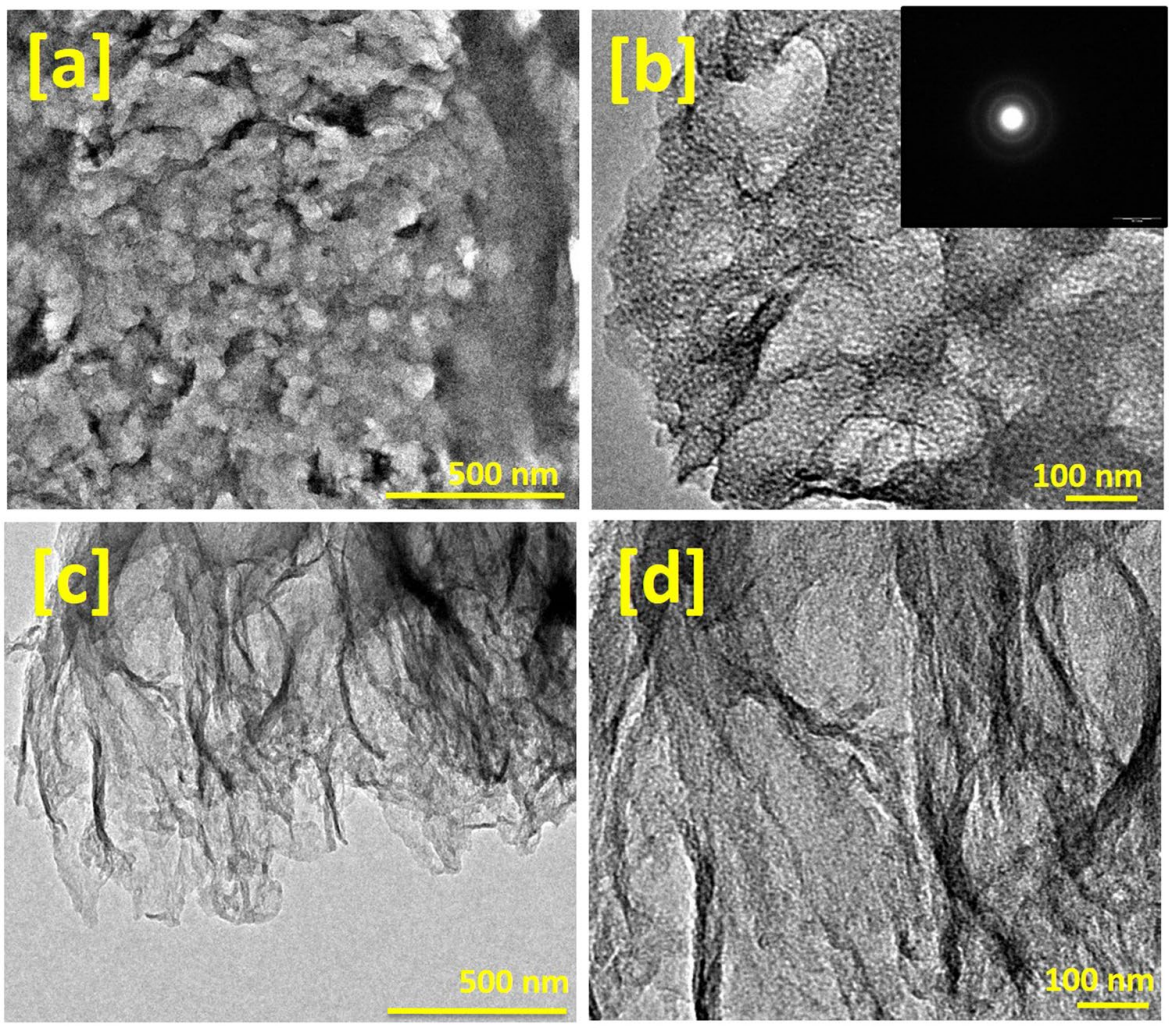
TEM images of the carbonized jute at various magnifications, (**a**) represents the porous microstructure, (**b**) further magnified version of figure (**a**); inset shows electron diffraction pattern of carbonized jute, (**c**) represents jute carbonized porous structure composed graphitic thin sheets, and (**d**) represents the magnified view of figure (**c**), where porosity of carbonized jute can be easily seen.

The resulting porous and sheets-like structure provides high surface area to volume ratio and significantly contributes to adsorption-desorption process during electrochemical double layer formation in suitable electrolytes. As pointed out above, the carbonized jute showed the presence of graphitic phase which is a conducting phase of carbon and thus advantageous for application as electrode materials for supercapacitors. Thus, the combination of these two properties: the presence of graphitic phase and the highly porous structure, makes the jute-derived activated carbon very suitable for such applications. Further, Fig. 4 represents the typical TEM images of the carbonized jute at various magnifications. The TEM micrographs clearly reveal the porous microstructural features of carbonized jute, which is composed of several graphitic thin layers. The TEM results are in good agreement with our observed BET data.

FT-IR spectrum of starting jute showed presence of several different types of oxygen groups: carbonyl, alcohol, ether and ester. Thus, broad and strong peak at 3380 cm^−1^ corresponded to -O-H stretching from hydroxyl groups on the jute surface (Fig. S2, ESI), peaks around 2928 and 2880 cm^−1^ represented asymmetric and symmetric C-H stretching mode, respectively. FT-IR peaks around 1744 and 1592 cm^−1^ were assigned to C=O and C=C stretching, respectively. A relatively intense peak around 1037 cm^−1^ was assigned to the R-OH group^41^. In contrast to this, the carbonized jute showed only carbon characteristics peaks, confirming the disappearance of other functional groups after chemical activation. The absence of other functional groups is beneficial for supercapacitor applications since such groups would increase the electrical resistivity of carbon. In agreement with this, Guo *et al*. have also observed that most of the functional groups were removed during the KOH activation process of sulfonated pitch^36^.

Thermal decomposition behaviour of jute fibers in nitrogen atmosphere is shown in Fig. S3. It is attributed to the pyrolysis of hemicellulose, cellulose, and lignin, with the small peak in temperature range of 50–100 °C most likely due to physical dehydration. The decomposition step in the 50–325 °C range could be attributed to the pyrolysis of hemicellulose and the strong decomposition step in the range 320–400 °C to the pyrolysis of cellulose/lignin. The yield of the residue at 700 °C was 11%.

**Figure 5:**
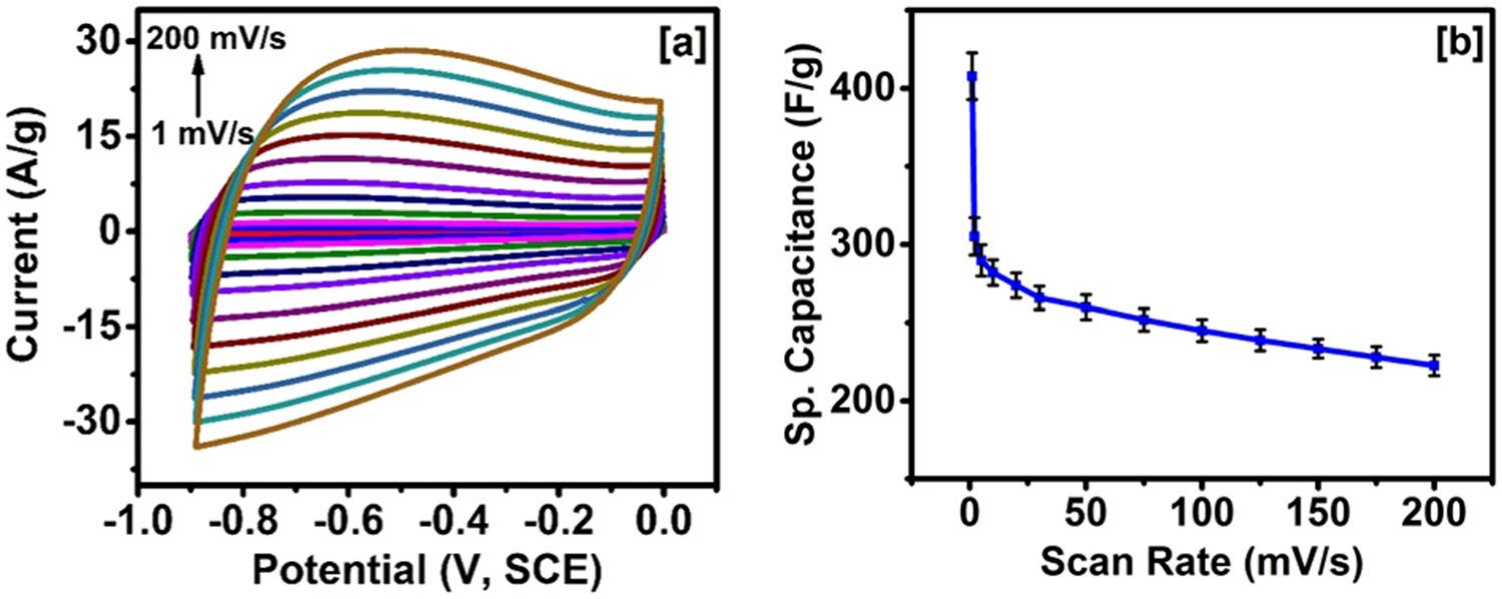
(**a**) CV curves of carbonized jute at various scan rate and (**b**) variation of specific capacitance versus scan rate.

The electrochemical properties of carbonized jute were studied using cyclic voltammetry (CV) and galvanostatic charge-discharge measurements. Figure 5a shows CV curves of the carbonized jute at various scan rates in 3 M KOH electrolyte. These curves have near rectangular shape, characteristic of a double layer capacitor. The absence of any redox peaks was in agreement with FT-IR results, and interestingly, the shape of the CV curves was retained even at higher scan rates, suggesting high-rate performance of the carbonized jute. Such performance even at a high scan rates can be attributed to porous structure which is expected to facilitate easy and smooth transport of electrolyte ions which prevents distortion of CV curves. The charge-storage mechanism for carbonized jute seems to be electrochemical double layer, as no redox peaks were observed in the CV curves, in agreement with the results reported for other examples of carbonized bio-mass^36, 42^. In electrochemical double layer charge storage mechanism, the electrolyte ions are reversibly adsorbing and desorbing on the surface, and the presence of pores in jute would be expected to significantly improve such a process by providing higher surface area and energy storage capacity. Since in electrochemical double layer charge-storage mechanism, only adsorption and desorption take place, there is no chemical reaction or redox process involved, stability and thus the performance could be very high.

Specific capacitance of the carbonized jute was calculated using the following equation^43^

2 $${C}_{sp}=\frac{{\int }^{}I.dV}{{\rm{\Delta }}V\times \vartheta \times m}$$

where *I* is the current, *ΔV* is the potential window, *ϑ* represents the scan rate and *m* is the mass of the carbonized jute fibers. Figure 5b, shows the change in specific capacitance as a function of scan rate. It can be seen from this figure that specific capacitance decreased with increasing scan rate, which could be due to insufficient time for the adsorption and desorption process. At higher scan rates, the concentration of the electrolyte ions near the electrode/electrolyte interface rises rapidly creating a situation in which there would be no sufficient time for the diffusion process to take place^44^. Specific capacitance of 408 ± 15 F/g was observed at 1 mV/s which decrease to 223 ± 6.6 F/g at 200 mV/s. Similar behaviour, i.e. decrease in specific capacitance with increase in scan rate, was observed for the waste-tire derived carbon-polymer composites^45^. A comparison of some important electrochemical data for other KOH activated bio-mass samples is given in Table 1.

The dependence of the voltammetric current on the scan rate of CV curves was studied to evaluate the reversibility and electrochemical properties of carbonized jute for supercapacitor application. Figure S4 (ESI), shows the variation of CV current as a function of scan rate. As seen in the graph, a linear dependence of the CV currents on the scan rate of CV measurements was observed. This suggest that the double layer charge-discharge currents are typically capacitive-like and a reversible charge-discharge response of the electric double layers.

**Figure 6:**
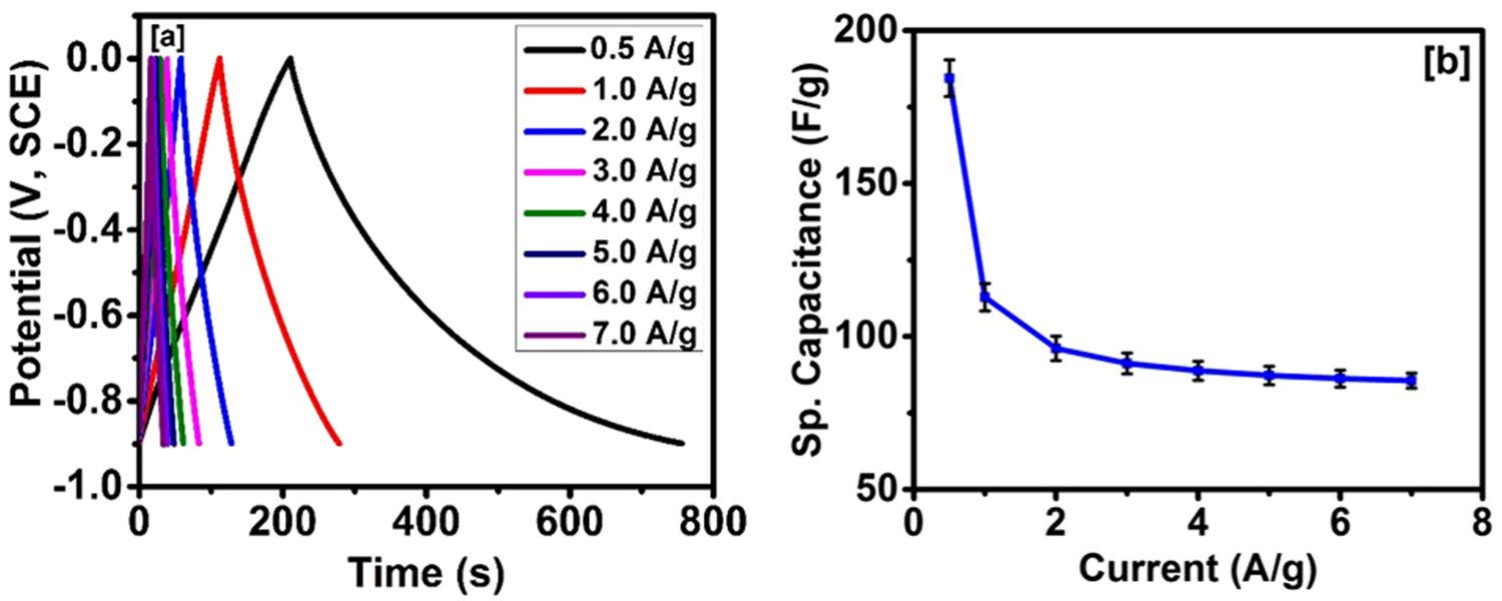
(**a**) Charge-discharge characteristics of carbonized jute and (**b**) variation of specific capacitance versus applied current.

The potential application of this carbonized jute as an electrode material for supercapacitor applications was further investigated using galvanostatic charge-discharge measurements. Figure 6, shows the galvanostatic charge-discharge characteristics of the carbonized jute. It is observed that the current has a significant effect on charge storage capacity of the carbonized jute. The discharge time decreased with increasing current density, which suggests a decrease in the charge storage capacity. The potential-time curves at various current densities were triangular in shape and symmetrical in nature, and this shape and nature were retained even at higher current densities, suggesting a high-rate performance. Small deviation (non-symmetrical) charge-discharge characteristics at low current density could be due to different rates of adsorption and desorption of electrolyte. Its seems that desorption of electrolyte (discharge) is a lower process. The charge storage capacity from the galvanostatic charge-discharge measurement was calculated using the following equation^54^:

3 $${C}_{sp}=\,\frac{I\times {\rm{\Delta }}t}{{\rm{\Delta }}V\times m}$$

where, *I* is the discharge current (*A*), Δ*t* is the discharge time (s), Δ*V* is the potential window (V) and *m* is the mass (g) of the carbonized jute. Figure 6b shows the effect of applied current on specific capacitance of the carbonized jute. As seen in Fig. 6b, decrease in the specific capacitance with increasing current density could be due to the diffusion limited process. At higher current density, the electrolyte ions don’t get sufficient time for the diffusion into the inner pores and thus provides lower capacitance^55^. Qian *et al*. have also observed sharp decrease in specific capacitance with increase in discharge current density for the activated carbon produced from human hair^6^. Table 1 compares electrochemical properties of different carbon materials prepared from natural materials and activated using KOH.

**Figure 7:**
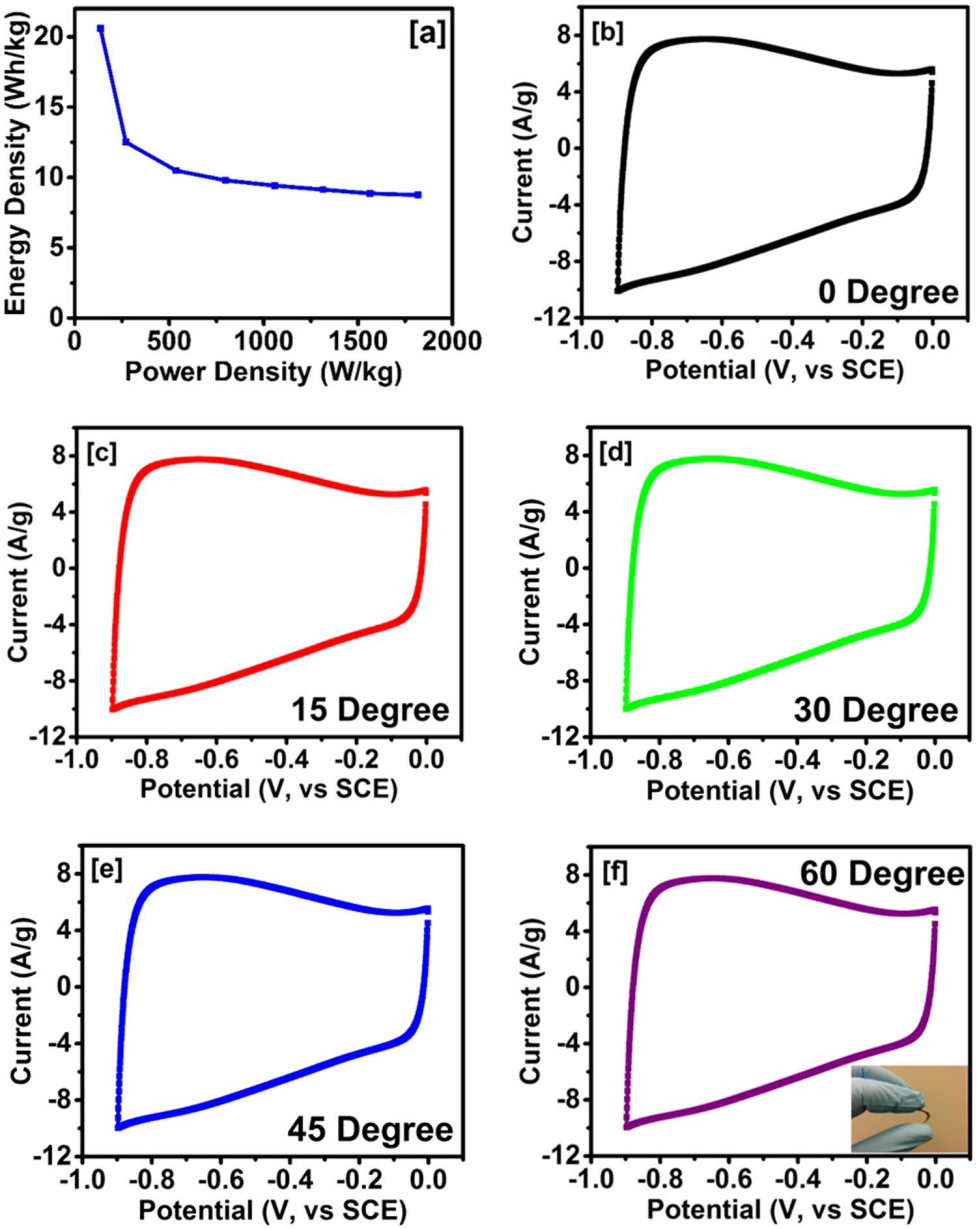
(**a**) Variation of power density versus energy density and (**b–f**) flexibility test of carbonized jute.

Energy and power densities are two important parameters that determine the performance of supercapacitor materials, and for carbonized jute we calculated them using the following expressions^56^:

4 $$E\,(\frac{Wh}{kg})=\frac{{C}_{sp}\times {\rm{\Delta }}{V}^{2}}{7.2}$$

5 $$P\,(\frac{W}{kg})=\frac{E\times 3600}{t}$$

where *C*_*sp*_ (F/g) is the specific capacitance calculated from galvanostatic charge-discharge measurements, *ΔV* (V) is the potential window and *t* (s) is the discharge time. Figure 7a, shows the Ragone plot determined from galvanostatic charge-discharge measurements. Carbonized jute showed the highest energy density of 21 Wh/kg whereas the highest power density was calculated to be 1.82 kW/kg. Qu *et al*. reported an energy density of 5.3 Wh/kg for the carbonized corncob in an alkaline medium^26^, while for KOH activated sunflower seed, an energy density and power density of 4.8 Wh/kg and 2.4 kW/kg, respectively were observed^40^.

**Figure 8:**
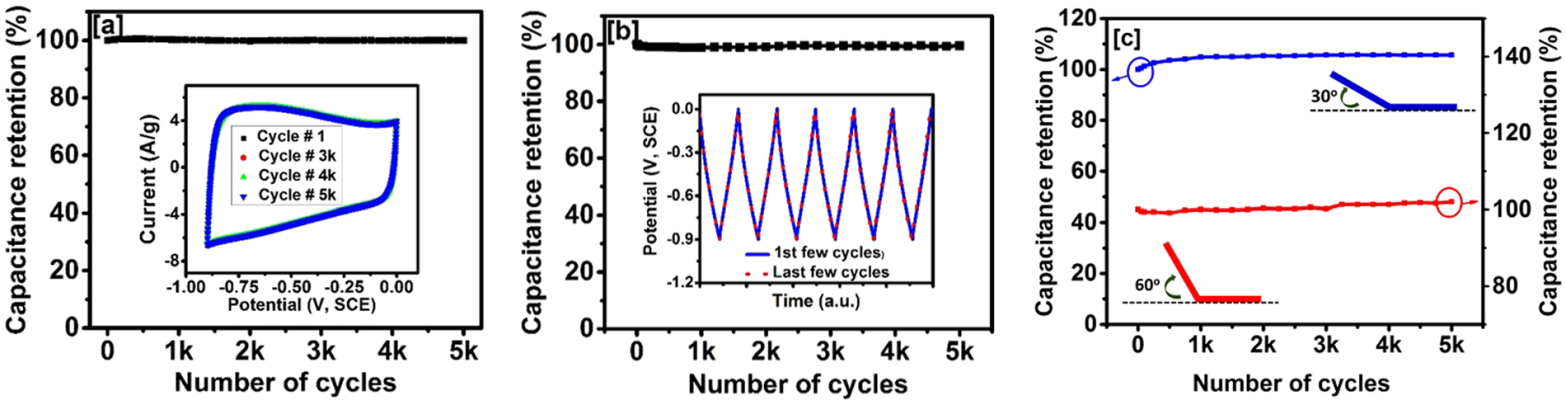
(**a**) Capacitance retention verse number of CV cycles, inset figure shows CV curves at various cycles (**b**) capacitance retention verse number of charge-discharge cycles, inset figure shows potential verses time plot for first and last few cycles of charge-discharge, and (**c**) capacitance retention verse number of charge-discharge cycles at various bending angles.

To estimate potential application of carbonized jute in flexible energy storage devices, CV curves were measured in the potential range of 0 to −0.9 V (vs, SCE) at various bending angles (Fig. 7b). It was found that the shape and area of the CV curve did not change with bending angle, suggesting that the carbonized jute could be used for flexible devices. Furthermore, long term electrochemical stability of carbonized jute electrodes was evaluated using both cyclic voltammetry and galvanostatic charge-discharge methods. Figure 8a shows the performance of the jute electrode as a function of the number of CV cycles. As seen in the figure, carbonized jute showed exceptional cyclic stability over several thousand cycles. The inset of Fig. 8a shows the cyclic voltammogram at various CV scans. The shape and size of the CV curves were identical even after 5,000 cycles which confirm the electrochemical stability of the carbonized jute. The stability of the carbonized jute over 5,000 galvanostatic charge-discharge cycles is shown in Fig. 8b. The results are very similar to what we observed using cyclic voltammetry stability test. For clarity, the inset of Fig. 8b shows potential versus time curves for first and last few cycles of galvanostatic charge-discharge measurements. The exact overlap of first few and last few cycles of potential versus time curves indicates that there was no degradation in the charge storage capacity, indicating its 100% retention. For comparison, Zhao *et al*. observed 86% capacitance retention after 900 cycles for potato starch based activated carbon^42^, while chemically activated tea leaves showed a capacitive retention of 92% after 2,000 cycles of charge discharge measurements^18^. In our study on the recycled jute, we have observed ~100% retention in charge storage capacity after 5,000 cycles of CV and charge-discharge measurements. The stability test of the carbonized jute was further investigated at various bending angles. Figure 8c shows the stability performance of the carbonized jute at various bending angles. As seen in the graph, the carbonized jute showed about 100% retention of charge storage capacity at different bending angles. The flexibility and stability tests on carbonized jute suggest that carbonized jute is very suitable for fabricating flexible and high performance energy storage devices.

In addition to the comparison with other bio-based carbons shown in Table 1, the electrochemical properties of carbonized jute were also compared to other types of carbon such as carbon nanotubes (CNT), multiwall CNT (MWCNT), graphene oxide (GO), reduced GO (rGO) and their composites with metal oxide are given. Sevilla *et al*. modified the surface of CNTs with N-doped carbon and observed an improvement in specific capacitance of CNT^57^. CNT showed a specific capacitance of about 21 F/g which increased 2- to 4- fold after surface modification with N-doped carbon. Polypyrrole-coated multiwalled carbon nanotubes (PPy-MWCNT) were used for the fabrication of activated carbon-coated MWCNT doped with nitrogen (N-ACMWCNT)^58^. The N-AC-MWCNT electrodes showed the maximum specific capacitance of about 103 F/g at a scan rate of 2 mV/s with almost 98% retention after 1,000 cycles of study. Composite of graphene oxide and polypyrrole (GO-PPy) showed that electrochemical properties of the composites increased with increase in GO concentration upto 10 wt% and then decreased with further addition of GO^59^. The maximum specific capacitance of 333 F/g was observed with about 86% retention in energy storage capacity. Table 1S compare the electrochemical properties of these other types of carbon.

**Figure 9:**
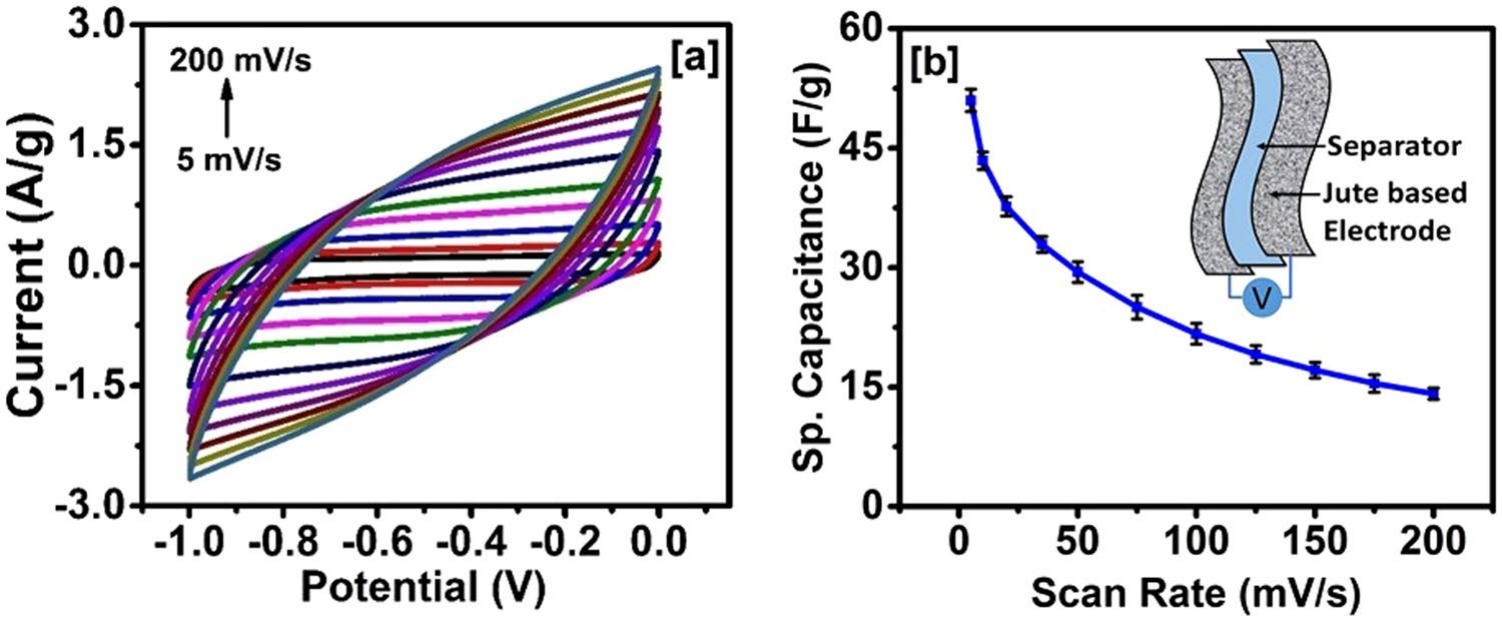
(**a**) CV curves at various scan rates and (**b**) variation of specific capacitance versus scan rate for the supercapacitor device made using carbonized jute, inset of figure (**b**) shows schematics of the supercapacitor device.

To further investigate potential applications of carbonized jute, we fabricated symmetrical supercapacitor device by sandwiching two jute electrodes separated by an ion transporting layer. The CV curves of this device at various scan rates are shown in Fig. 9a. The shape of the CV curves of the jute based supercapacitor device is identical in nature, even at higher scan rates, suggesting high-rate performance of the device. The variation of specific capacitance as a function of scan rate in in Fig. 9b, showed a decrease of specific capacitance with increasing scan rate, which could be due to insufficient time for the electrolyte ions to reach into the pores of carbonized jute and contribute to the charge storage process.

**Figure 10:**
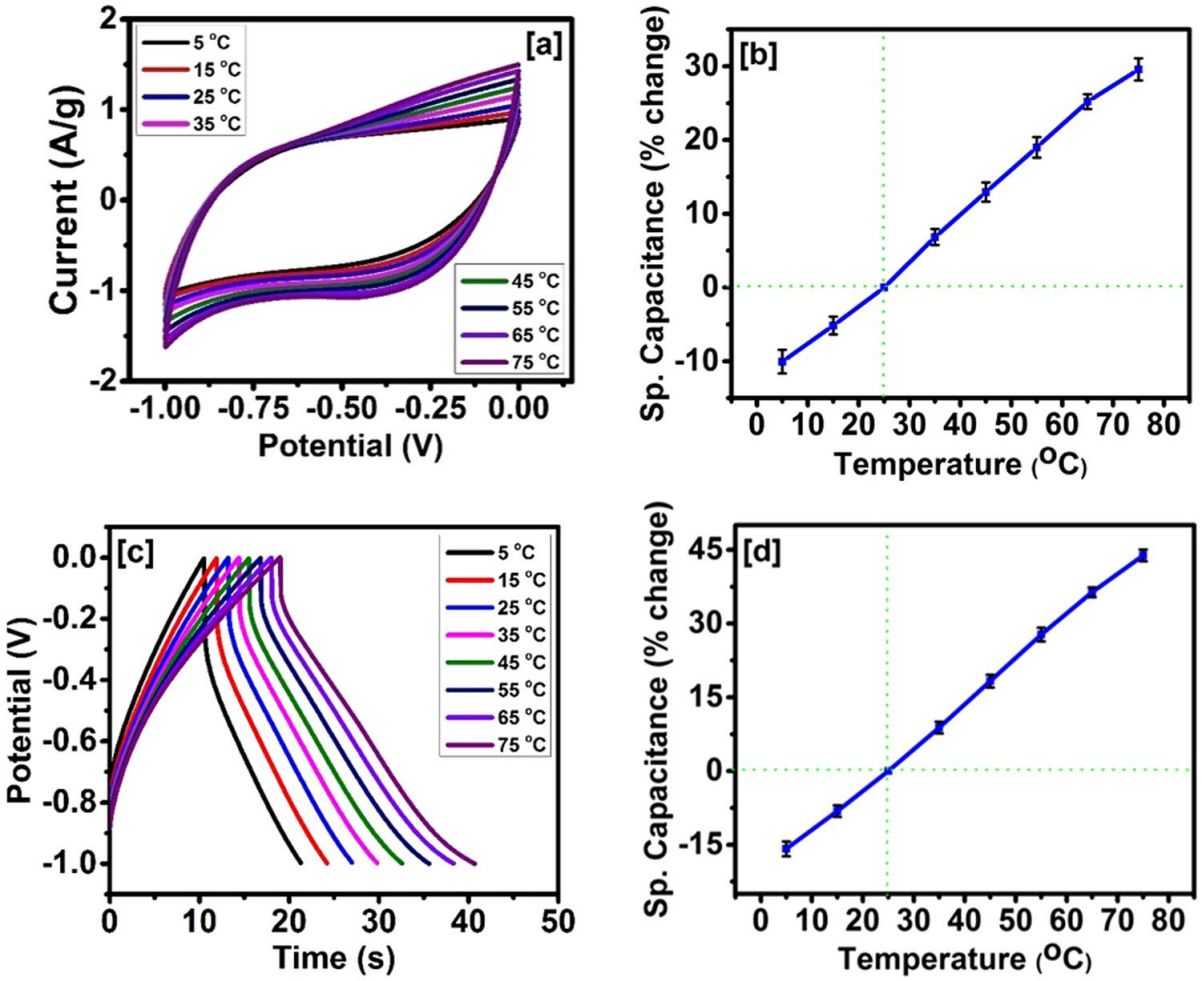
(**a**) CV curves at various temperatures at 50 mV/s, (**b**) % change in specific capacitance versus temperature, (**c**) charge discharge characteristics at various temperature at 1 mA, and (**d**) % change in specific capacitance versus temperature for the supercapacitor device made using carbonized jute.

After successful testing for the stability and flexibility of the carbonized jute fibers based electrodes, we investigated its performance at different temperatures. The cyclic voltammetry and galvanostatic charge-discharge measurements were performed as a function of temperature in the range of 5–75 °C. Figure 10a shows that the area under the CV curves increased with temperature, suggesting an improvement in the electrochemical charge storage capacity of the device. In addition, the shape of the curves was found to be identical, indicating electrochemical stability of the device even at higher temperature, with about 60% improvement in charge storage capacity on raising the temperature from 5 °C to 75 °C (Fig. 10b). The effect of temperature on the charge storage capacity was also examined using galvanostatic charge-discharge measurements. Figure 10c shows the charge-discharge (potential versus time) characteristics of the device at different temperatures. The discharge time increased with increase in temperature, suggesting that the device had by about 45% higher charge storage capacity increasing the temperature from room temperature to 75 °C (Fig. 10d).

The effect of temperature on the electrochemical behaviour of the supercapacitor was further investigated using electrochemical impedance spectroscopy. The variation of real and imaginary impedance of the supercapacitor device at various temperatures is shown in Fig. S5a (ESI). The real and imaginary impedance of the device, and the equivalent series resistance (ESR) decreased with increasing temperature. This decrease in ESR has a very positive effect on the capacitance of the supercapacitor device, and it could be due to the enhanced mobility of the ions through the electrolyte which increases the conductivity of the latter^60^. Total impedance of the device also decreased with increasing temperature and frequency (Fig. S5b).

## Conclusions

We developed a new type of carbon-based electrodes for supercapacitor by carbonizing abundantly available jute fibres followed by their chemical surface activation. As confirmed by XRD, Raman, BET and TEM analysis, the carbonized jute fibers were graphitic in nature and contained micropores. The electrochemical studies of electrodes based on this carbonized jute showed the highest specific capacitance of about 408 F/g in KOH electrolyte, very high power and energy densities with excellent cyclic stability and flexibility. In addition, a supercapacitor device fabricated using this carbonized jute showed a promising specific capacitance of about 51 F/g and temperature dependent performance with over 60% improved specific capacitance at 75 °C.

## Electronic supplementary material

SUPPLEMENTARY INFO
