# Supplementary material for: High-Performance Flexible Supercapacitors obtained via Recycled Jute: Bio-Waste to Energy Storage Approach
Source: Sci Rep. 2017 Apr 26;7:1174. doi: 10.1038/s41598-017-01319-w (PMC5430782; doi:10.1038/s41598-017-01319-w)
Supplement: Supplementary file 1 — SUPPLEMENTARY INFO [file 41598_2017_1319_MOESM1_ESM.pdf]

Electronic Supplementary Information (ESI)

High-Performance Flexible Supercapacitors obtained via Recycled Jute: Bio-Waste to Energy Storage Approach

Camila Zequine[1], C. K. Ranaweera[1], Z. Wang[1], Petar R. Dvornic[1], P. K. Kahol[2], Sweta Singh[3], Prashant Tripathi[3], O.N. Srivastava[3], Satbir Singh[4], Bipin Kumar Gupta[4], Gautam Gupta[5], Ram K. Gupta[1]*

[1]Department of Chemistry, Pittsburg State University, 1701 S. Broadway, Pittsburg, Kansas 66762, USA
[2]Department of Physics, Pittsburg State University, 1701 S. Broadway, Pittsburg, Kansas 66762, USA
[3]Department of Physics, Banaras Hindu University, Varanasi, Uttar Pradesh, 221004, India
[4]CSIR -National Physical Laboratory, Dr. K.S. Krishnan Road, New Delhi 110012, India
[5]Materials Physics and Applications (MPA-11), Los Alamos National Laboratory, Los Alamos, New Mexico 87545, USA

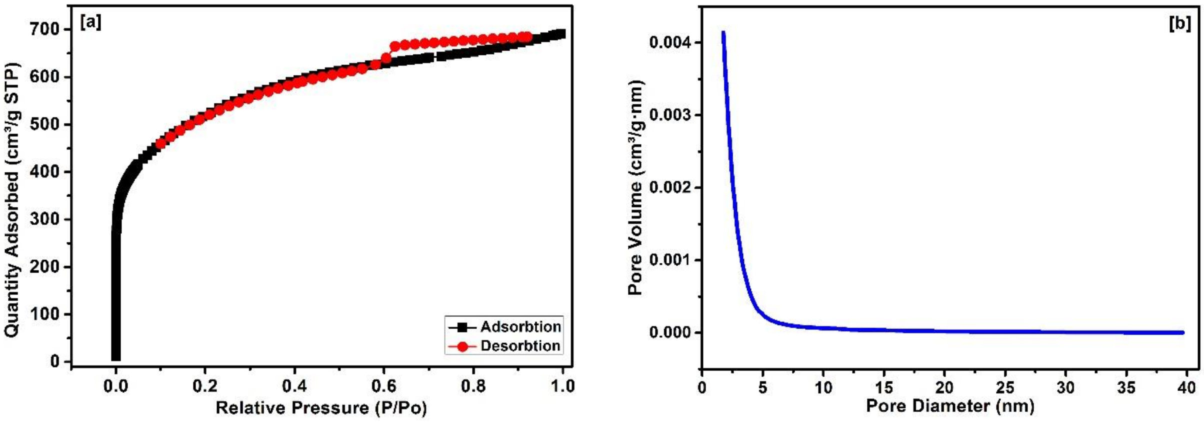

Fig. S1: (a) Adsorption/desorption isotherm of N_2 and (b) pore size distribution from BJH adsorption in N_2 for carbonized jute.

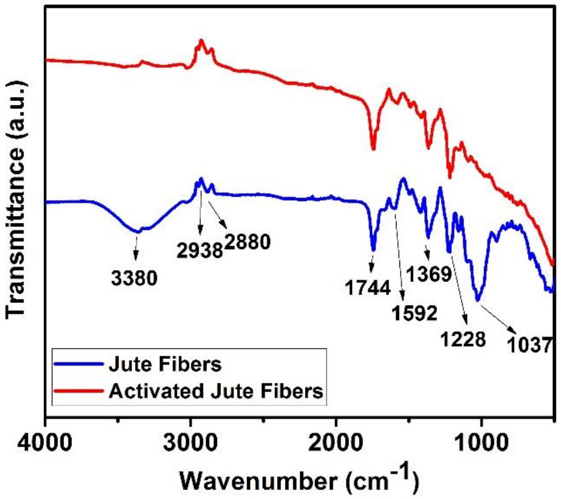

Figure S2. FT-IR spectrum of the carbonized jute fibers.

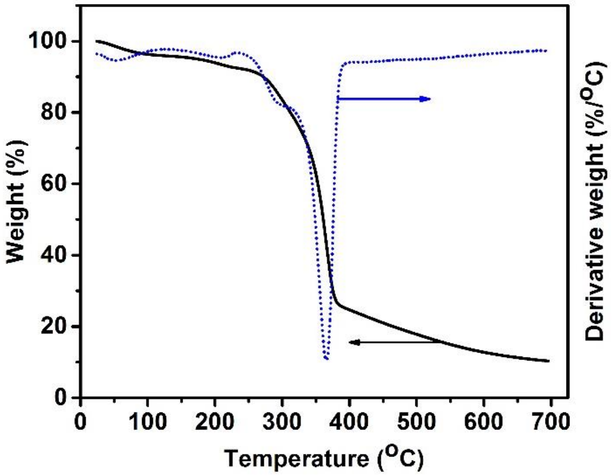

Figure S3. TGA curve of recycled jute fibers.

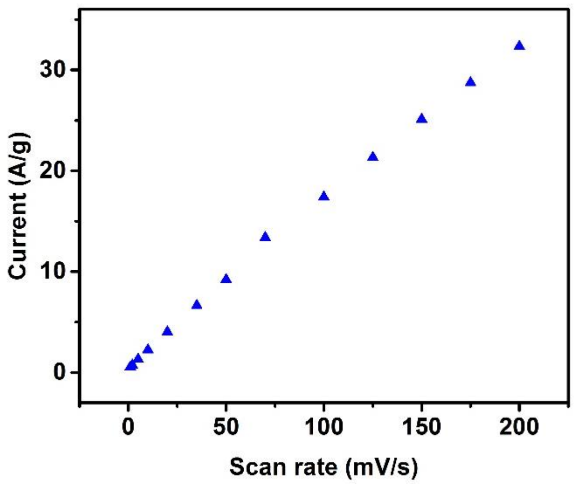

Figure S4. Dependence of voltammetric current on scan rate of CV measurements.

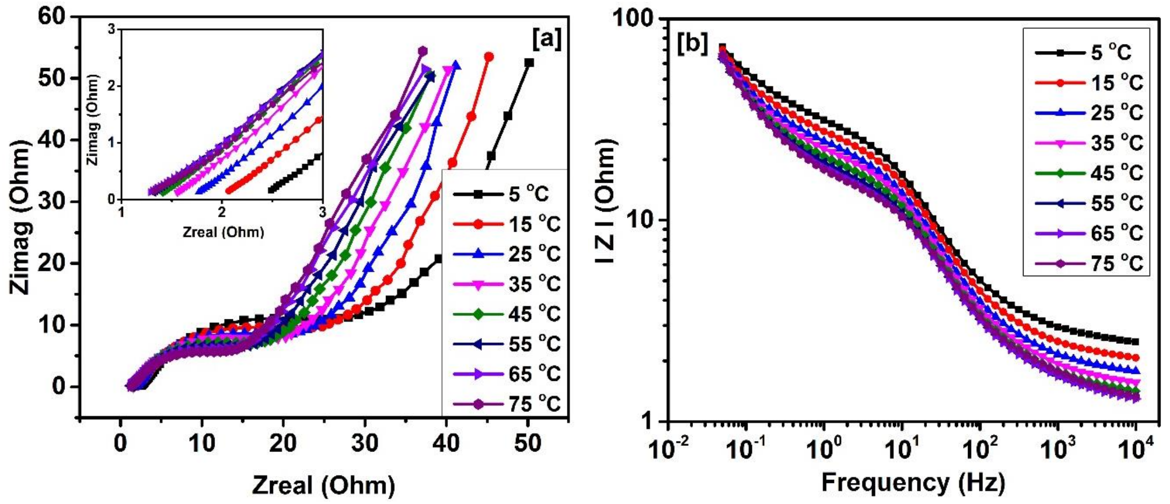

Figure S5: (a) variation of Z_{re} and I_{img} and (b) variation of impedance as a function of frequency for the supercapacitor device at various temperature for the supercapacitor device made using carbonized jute.

Table 1S: Electrochemical properties of carbon based materials and their composites.

Material	Max. Capacitance (F/g)	Electrochemical testing performed under	Reference
CNT	21	1 mV/s, 1 M H_2SO_4	1
Nitrogen doped activated carbon-MWCNT	103	2 mV/s, 0.5M Na_2SO_4	2
CNT-Graphene	199	0.5 A/g, 1-ethyl-3-methylimidazolium tetrafluoroborate	3
Graphene microsphere	151	10 mV/s, 1M H_2SO_4	4
N-doped graphene	205	0.1 A/g, 6M KOH	5
B-doped rGO	193	0.1 A/g, 6M KOH	6
Graphene/Polyaniline	233	2 mV/s, 1M H_2SO_4	7
Graphene–TiO_2	165	5Mv/s, 1M Na_2SO_4	8
rGO/CNTs/MnO_2	319	0.5 A/g, 1M Na_2SO_4	9
rGO/Fe_3O_4	220	0.5 A/g, 1M KOH	10
GO/MnO_2	328	10 Mv/s, 1M Na_2SO_4	11
Graphene-tungsten oxides	144	0.1A/g, 1MH_2SO_4	12
rGO/Co_3O_4	472	2mV/s, 2M KOH	13

References:

1 Sevilla, M., Yu, L., Zhao, L., Ania, C. O. & Titiricic, M.-M. Surface Modification of CNTs with N-Doped Carbon: An Effective Way of Enhancing Their Performance in Supercapacitors. *ACS Sustainable Chemistry & Engineering* **2**, 1049-1055, (2014).

2 Shi, K., Ren, M. & Zhitomirsky, I. Activated Carbon-Coated Carbon Nanotubes for Energy Storage in Supercapacitors and Capacitive Water Purification. *ACS Sustainable Chemistry & Engineering* **2**, 1289-1298, (2014).

3 Pham, D. T. *et al.* Carbon Nanotube-Bridged Graphene 3D Building Blocks for Ultrafast Compact Supercapacitors. *ACS Nano* **9**, 2018-2027, (2015).

4 Park, S.-H. *et al.* Spray-Assisted Deep-Frying Process for the In Situ Spherical Assembly of Graphene for Energy-Storage Devices. *Chem. Mater.* **27**, 457-465, (2015).

5 Liu, Y.-Z. *et al.* Easy one-step synthesis of N-doped graphene for supercapacitors. *Energy Storage Materials* **2**, 69-75, (2016).

6 Han, J. *et al.* Generation of B-Doped Graphene Nanoplatelets Using a Solution Process and Their Supercapacitor Applications. *ACS Nano* **7**, 19-26, (2013).

7 Wang, D.-W. *et al.* Fabrication of Graphene/Polyaniline Composite Paper via In Situ Anodic Electropolymerization for High-Performance Flexible Electrode. *ACS Nano* **3**, 1745-1752, (2009).

8 Ramadoss, A. & Kim, S. J. Improved activity of a graphene–TiO2 hybrid electrode in an electrochemical supercapacitor. *Carbon* **63**, 434-445, (2013).

9 Jiang, H., Dai, Y., Hu, Y., Chen, W. & Li, C. Nanostructured Ternary Nanocomposite of rGO/CNTs/MnO2 for High-Rate Supercapacitors. *ACS Sustainable Chemistry & Engineering* **2**, 70-74, (2014).

10 Wang, Q., Jiao, L., Du, H., Wang, Y. & Yuan, H. Fe3O4 nanoparticles grown on graphene as advanced electrode materials for supercapacitors. *J. Power Sources* **245**, 101-106, (2014).

11 Kim, M., Hwang, Y. & Kim, J. Graphene/MnO2-based composites reduced via different chemical agents for supercapacitors. *J. Power Sources* **239**, 225-233, (2013).

12 Cai, Y. *et al.* Graphene nanosheets-tungsten oxides composite for supercapacitor electrode. *Ceram. Int.* **40**, 4109-4116, (2014).

13 Xiang, C., Li, M., Zhi, M., Manivannan, A. & Wu, N. A reduced graphene oxide/Co3O4 composite for supercapacitor electrode. *J. Power Sources* **226**, 65-70, (2013).
